# Vinorelbine induced perforation of a metastatic gastric lesion

**DOI:** 10.1007/s11845-016-1536-1

**Published:** 2016-12-30

**Authors:** W. J. Mullally, C. B. O’Súilleabháin, C. Brady, S. O’Reilly

**Affiliations:** 1 Department of Medical Oncology, Cork University Hospital, Wilton Rd, Cork, Ireland; 2 Hepatobiliary Pancreas Unit, Mercy University Hospital, Grenville Place, Cork, Ireland; 3 Kells, Bishopstown Avenue West, Model Farm Rd, Cork, Ireland

**Keywords:** Metastatic breast cancer, Vinorelbine, Gastric perforation, Gastric side effects

## Abstract

**Background:**

Breast carcinoma metastasis to the gastrointestinal tract is rare and more frequently associated with lobular than ductal carcinoma (Borst and Ingold, Surg 114(4):637–641 [1]). The purpose of this article is to present a case based review of a unique gastrointestinal metastasis and literature review.

**Methods:**

A 46 year old lady with metastatic invasive ductal breast cancer was admitted to A&E with sudden onset of epigastric and left shoulder pain. She completed the first cycle of capecitabine/vinorelbine 1 week previously. Clinical examination revealed a tender epigastrium with rigidity in the upper abdomen. Free air under the diaphragm and a positive Rigler’s sign was radiologically identified. A laparoscopy demonstrated a fibrinous exudate in the left upper quadrant consistent with a walled off lesser curvature gastric perforation. A subsequent oesophagogastroduodenoscopy (OGD) demonstrated a healed gastric ulcer of benign appearance; however the pathology confirmed metastatic breast carcinoma.

**Results:**

Literature review confirmed no previously reported cases of vinorelbine induced gastric perforation. Four cases of metastatic breast cancer with gastric metastasis presenting with perforation were identified; three of these cases (Fra et al., Presse Med 25(26):1215 (1996) [2], Solis-Caxaj et al., Gastroenterol Clin Biol 28(1):91–92 (2004) [3], Ghosn et al., Bull Cancer 78(11):1071–1073 (1991) [4]), were in the French medical literature, including one male patient (Fra et al., Presse Med 25(26):1215 (1996) [2]) and at least one ductal breast carcinoma (Solis-Caxaj et al., Gastroenterol Clin Biol 28(1):91–92 (2004) [3]). The fourth case (van Geel et al., Ned Tijdschr Geneeskd 144(37):1761–1763 (2000) [5]), was in the Dutch medical literature and a lobular breast carcinoma.

**Conclusion:**

This case represents a rare complication of breast cancer chemotherapy, the subsequent significant benefit the patient received from treatment is consistent with the chemosensitivity to therapy that also resulted in gastric perforation. Five years after gastric perforation she resumed palliative chemotherapy after progression on sequential hormonal therapies.

## Introduction

Breast cancer is the most common female cancer with a lifetime incidence of 10% [6]. Frequent metastatic sites include lung, brain, liver, bone, soft tissue and adrenal glands. However metastasis to the gastrointestinal tract is rare [7], with gastric involvement rarer. The presenting symptoms in patients are similar to primary gastric carcinoma and can include epigastric pain, early satiety and weight loss. This report reviews a metastatic breast cancer case over a 14 year period and includes a rare chemotherapy complication.

## Materials and methods

A computerised literature review was completed using PubMed including the following keywords: metastatic breast cancer, vinorelbine, gastric perforation & gastric side effects. Toxnet case reports were also accessed to review vinorelbine safety profile.

## Case

A 38 year old lady was diagnosed with a invasive ductal carcinoma [IDC]. A two year history of an intermittent discharge which subsequently developed into a lateral lump was noted. A left mastectomy was performed, pathology demonstrated a 1.8 cm node negative [pT_1c_N_0_M_x_] lesion. On immunohistochemistry the tumour was positive for oestrogen receptor and negative for progesterone receptor and human epidermal growth factor receptor 2 [HER2]. Adjuvant chemotherapy, hormonal therapy and radiotherapy were administered subsequently.

Six year later, right neuropathic shoulder pain developed. X-ray imaging confirmed incidental pulmonary nodules. A subsequent CT-TAP confirmed right hepatic lobe lesions. A lung biopsy demonstrated metastatic breast cancer. Palliative Docetaxel chemotherapy and subsequently hormonal therapy was commenced.

**Fig. 1:**
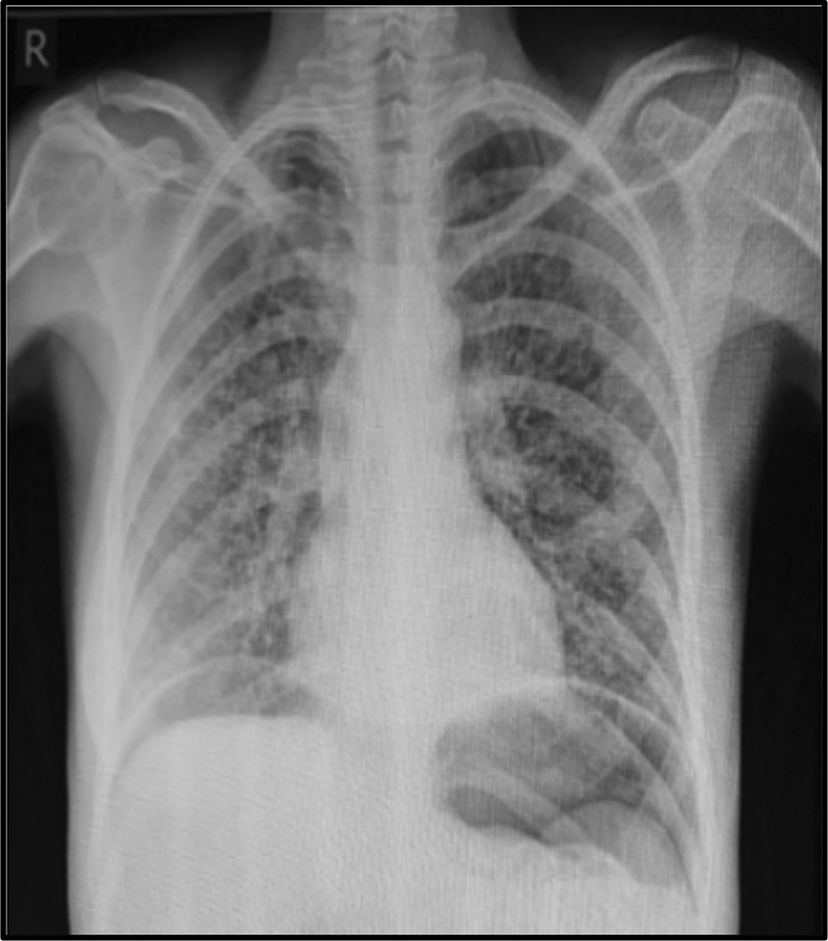
CXR with bilateral pneumoperitoneum

**Fig. 2:**
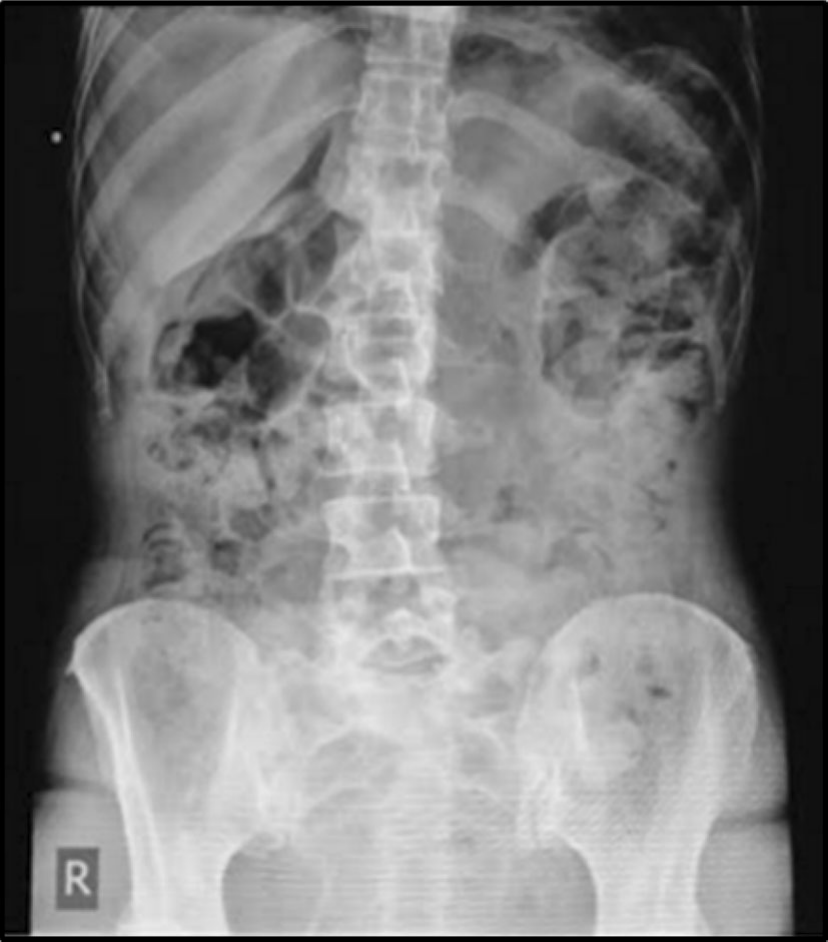
PFA with Rigler’s Sign

Two years later [now 46 years old] progressive disease was noted in lung, liver and bone. Capecitabine and Vinorelbine chemotherapy was commenced. Eight days after initiation she was admitted to A&E with sudden onset epigastric and left shoulder pain. Clinical examination revealed a tender epigastrium with rigidity in the upper abdomen. Radiological imaging demonstrated free air under the diaphragm [Fig. 1] with a positive Rigler’s sign [Fig. 2].

**Fig. 3:**
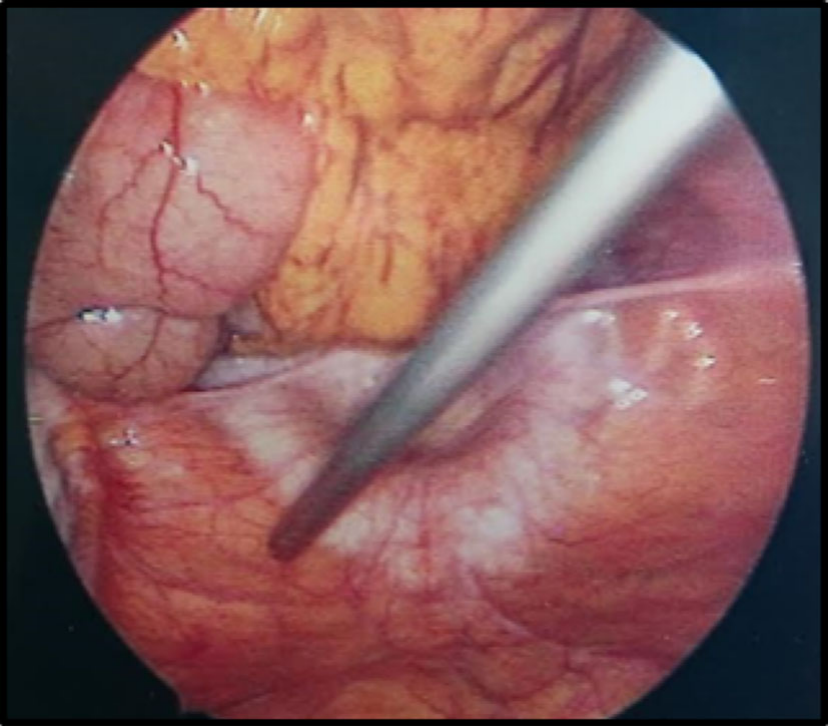
Fibrinous exudate

**Fig. 4:**
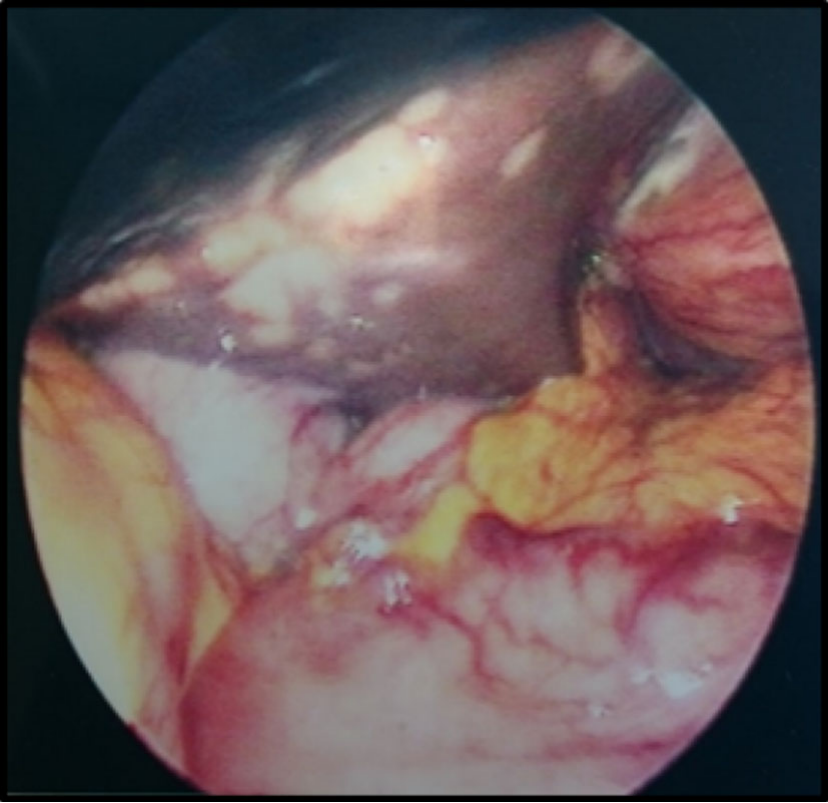
Hepatic metastasis

**Fig. 5:**
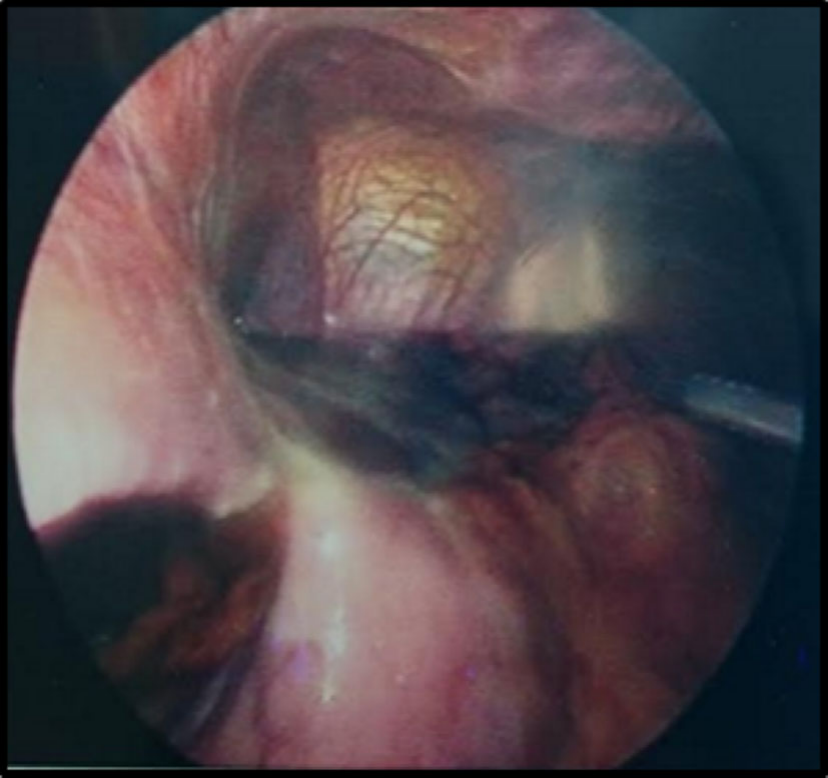
Pelvic free fluid

A laparoscopy demonstrated a fibrinous exudate in the left upper quadrant consistent with a walled off lesser curvature gastric perforation [Fig. 3]. Multiple hepatic metastases [Fig. 4] were identified. A pelvic wash out removed a large volume of free pelvic fluid [Fig. 5]. The ulcer was not disturbed.

A subsequent OGD completed 16 days later demonstrated a healed gastric ulcer of benign appearance. Pathology analysis demonstrated tiny microscopic foci of ductal carcinoma proximal to the muscularis mucosa consistent with breast metastasis. Chemotherapy was reinitiated.

Three months later this lady represented with a three day history of lower abdominal pain, two day history of no bowel motion with a single episode of non-feculent vomitus. Radiological imaging demonstrated a mild reduction in the size of her metastatic lesions however it was unable to precisely identify the source of the small bowel obstruction. A Gastrograffin swallow and meal confirmed gastric distension with abrupt transition at the first part of the duodenum [D1] with a sliver of contrast passing beyond D1. A palliative laparoscopic gastroduodenectomy was completed. This admission was further complicated by *Closteridium difficile* and *Klebsiella pneumonia*. Hormonal therapy was reintroduced and continued for a further five years until disease progression in lung and liver metastasis and palliative chemotherapy has been reinitiated.

## Results

This patient developed gastric metastasis to the lesser curvature eight years following her primary breast cancer. This lesion perforated owing to its exquisite chemosensitivity. Similarly 3 months later the metastatic lesion at pylorus/D1 was also chemo sensitive; to such an extent its rapid regression induced fibrosis resulting in a small bowel obstruction at D1.

Literature review of PubMed and Toxnet confirmed no previously reported cases of vinorelbine induced gastric perforation. Four cases (Non-English literature) of metastatic breast cancer with gastric metastasis presenting with perforation were identified. Three of these cases [2–4], were in French, including one male [2], patient and at least one ductal primary breast carcinoma [3]. The fourth case [5], was in Dutch and involved a lobular primary breast carcinoma.

Further keyword searches included vinorelbine, side effects and metastatic breast cancer yielding 316 articles. However upon further review of the abstracts less than ten suitable articles (majority being case reports) were identified. The safety profile of vinorelbine was well tolerated; though rare cases of paralytic ileus/intestinal obstruction, typhlitis, reversible posterior leukoencephalopathy syndrome (RPLS), fatal *Clostridium difficle* infection, SIADH, hand & foot syndrome, pancreatitis and pulmonary oedema were reported.

## Discussion

The patient in this report was diagnosed with metastatic breast cancer eight years after initial presentation. Metastatic breast cancer to the gastrointestinal tract is rare [1], and tumour registry data collected over 18 years with 2605 [1], cases reports demonstrates prevalence of 4.5% ILC and 0.2% IDC. Metastatic gastric involvement is rarer and Taal et al. [8], reported 83% of gastric metastatic cases involved the invasive lobular carcinoma (ILC) sub-type. The exact reasoning for this is unknown and postulates include reduced e-cadherins expressions associated with ILC tropism.

This symptoms associated with gastric metastasis are non-specific including nausea, vomiting abdominal pain [9], and early satiety. However in this case, there were none. The four day history of moderate reflux occurred within days of commencing cycle 1 of capecitabine/vinorelbine and was associated with the ruptured metastatic ulcer. The clinical signs were suggestive of a ruptured duodenal ulcer. Accordingly this provides a significant challenge in identifying such rare metastasis occurrences. However radiological imaging suggested a visceral perforation with laparoscopic laparotomy confirmation. A walled off ruptured ulcer on the lesser gastric curvature was an unexpected finding and remained undisturbed. This warranted conservative management during her admission. Such a rupture was responsible for the free pelvic fluid collection. The physical appearance of this ulcer upon OGD suggested a benign appearance and it was the histology which confirmed metastatic gastric involvement. The most common histology is linitis plastic with diffuse infiltration of the submucosa and muscularis propria; less commonly discrete nodules or external compression may occur [7]. This poses a challenge in distinguishing a primary adenocarcinoma from metastatic involvement. Therefore a comparison between the primary breast cancer pathology and gastric biopsy is essential to resolve any uncertainty. Immunohistochemical analysis also plays an important role. The absence of e-cadherin expression and ERα positivity in a gastric biopsy can establish metastatic breast cancer in ER positive patients [10]. Positive staining for GCDFP-15 (gross cystic disease fluid protein-15) has a sensitivity of 55–76% and specificity of 95–100% [11]. Immunohistochemistry was not required in this case as the diagnosis was confirmed from the histology.

This case also highlights a long lag time (of over eight years) between the initial breast cancer diagnosis and gastric metastasis. Pectasides et al. [12], identified eight breast cancer patients from their database (1995–2008) with median time to gastric involvement of 41 months. Two of these patients had IDC with gastric metastasis after 18 and 38 months, which is much earlier than this case. The median survival time was 11 months [range 1–44+ months] and within the IDC subset it was 23 and 4 months, respectively. McLemore et al. [13], identified 73 patients with gastrointestinal metastasis and carcinomatosis in which 28% had gastric involvement (64% ILC). The median survival time was 28 months. Furthermore surgical interventions did not significantly improve overall survival time. This contrasts with this case where the patient is still alive after 5½ years and did benefit from palliative gastroduodenectomy.

In conclusion it is essential to consider gastric metastasis in breast cancer patients, even though a second primary is more frequently observed. Histology, immunohistochemistry confirms the diagnosis when radiological/interventional investigations present any doubt. This is an unusual case where the chemotherapy, vinorelbine, within the first week of cycle one induced perforation of the metastatic gastric lesion. Ironically this is a significant reason as to why this lady is still alive almost 14 years after her initial breast cancer diagnosis.
